# Testimony of Experience: Docta Ignorantia and the Philadelphia Association Communities

**DOI:** 10.1192/pb.bp.114.048561

**Published:** 2015-06

**Authors:** Duncan Double

R. D. Laing and others founded the Philadelphia Association (PA) in 1965. The PA provides community households where people with emotional difficulties can live with others. The first ‘official’ community was the infamous Kingsley Hall, a ‘counterculture’ centre in the East End of London, which after 5 years was largely trashed and uninhabitable. In retrospect, Laing admitted that it was not a ‘roaring success’ (*Conversations with R. D. Laing*, B. Mullan). Nonetheless, despite the commonly perceived demise of ‘anti-psychiatry’, with which Laing was associated, the PA has survived nearly 50 years and still runs two community houses. In this book, Bruce Scott, a member of the PA, where he did his psychoanalytic psychotherapy training, offers the testimonies of 14 people who have lived in a PA household. These were obtained mostly by face-to-face interviews or by questionnaire.

Scott sees the PA communities as providing true asylum, in the sense of an ‘inviolable place’. There is no discussion, however, about whether such asylum is possible if the person is detained under the Mental Health Act 1983. My guess is Scott would say not. He makes a case for *docta ignorantia* or the doctrine of learned ignorance, a concept used by Nicolas Cusanus in the 15th century to recognise the limits of knowledge. For Scott, this is a path to health practised by the PA communities. However, there is little discussion about whether such neutrality is attainable. I am uncertain whether Scott’s search for an ‘anti-method’ is anything more than being pragmatic. The testimonies commonly mention the lack of structure in the households. I have no problem with mystery and perplexity and I totally agree with an anti-materialistic stance for dealing with mental distress. The PA rightly wants to avoid the objectification of people with mental health problems. Helping them find their own way is not easy.

This book describes the tension between ‘going to pieces’ and being helped to ‘come back together again’. Regression and psychosis can be mechanisms of healing and re-adaptation, as noted by Donald Winnicott among others. The PA continues to explore these areas, as does this book, but it may be increasingly difficult to find space for them in a bureaucratic society.

